# Long non-coding RNA Small Nucleolar RNA Host Gene 4 ameliorates cigarette smoke-induced proliferation, apoptosis, inflammation, and airway remodeling in alveolar epithelial cells through the modulation of the mitogen-activated protein kinase signaling pathway via the microRNA-409-3p/Four and a Half LIM Domains 1 axis

**DOI:** 10.1186/s40001-024-01872-x

**Published:** 2024-06-04

**Authors:** Meng Liu, JiGuang Meng, XuXin Chen, Fan Wang, ZhiHai Han

**Affiliations:** Department of Respiratory and Critical Care Medicine, The Sixth Medical Center of PLA General Hospital, Beijing, 100037 China

**Keywords:** Lnc SNHG4, miR-409-3p, FHL1, MAPK, COPD

## Abstract

The long non-coding RNA (lncRNA) Small Nucleolar RNA Host Gene 4 (SNHG4) has been demonstrated to be significantly downregulated in various inflammatory conditions, yet its role in chronic obstructive pulmonary disease (COPD) remains elusive. This study aims to elucidate the biological function of SNHG4 in COPD and to unveil its potential molecular targets. Our findings reveal that both SNHG4 and Four and a Half LIM Domains 1 (FHL1) were markedly downregulated in COPD, whereas microRNA-409-3p (miR-409-3p) was upregulated. Importantly, SNHG4 exhibited a negative correlation with inflammatory markers in patients with COPD, but a positive correlation with forced expiratory volume in 1s percentage (FEV1%). SNHG4 distinguished COPD patients from non-smokers with high sensitivity, specificity, and accuracy. Overexpression of SNHG4 ameliorated cigarette smoke extract (CSE)-mediated inflammation, apoptosis, oxidative stress, and airway remodeling in 16HBE bronchial epithelial cells. These beneficial effects of SNHG4 overexpression were reversed by the overexpression of miR-409-3p or the silencing of FHL1. Mechanistically, SNHG4 competitively bound to miR-409-3p, mediating the expression of FHL1, and consequently improving inflammation, apoptosis, oxidative stress, and airway remodeling in 16HBE cells. Additionally, SNHG4 regulated the miR-409-3p/FHL1 axis to inhibit the activation of the mitogen-activated protein kinase (MAPK) pathway induced by CSE. In a murine model of COPD, knockdown of SNHG4 exacerbated CSE-induced pulmonary inflammation, apoptosis, and oxidative stress. In summary, our data affirm that SNHG4 mitigates pulmonary inflammation, apoptosis, and oxidative damage mediated by COPD through the regulation of the miR-409-3p/FHL1 axis.

**Graphical Abstract:**

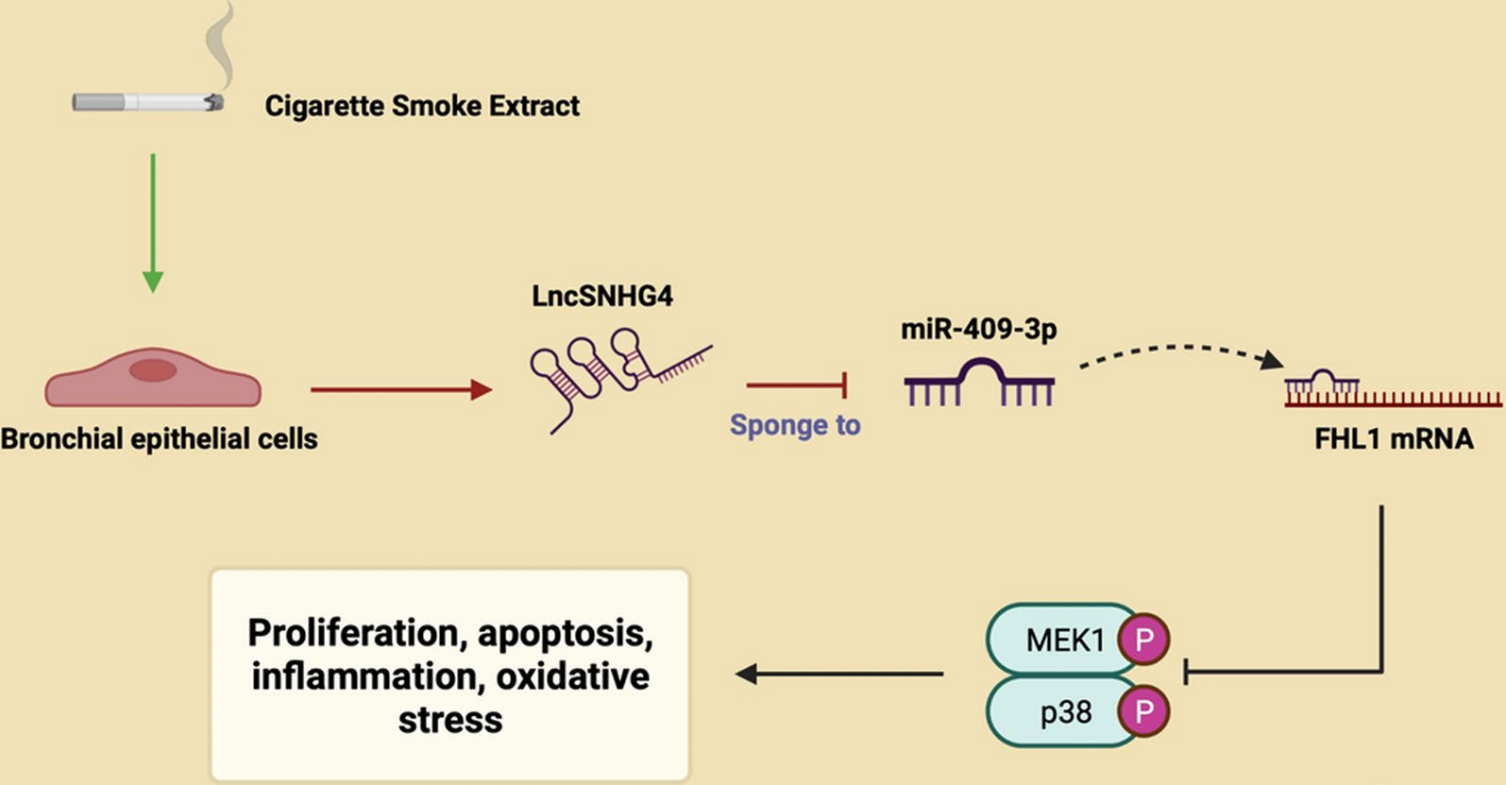

**Supplementary Information:**

The online version contains supplementary material available at 10.1186/s40001-024-01872-x.

## Highlights

1. SNHG4 and FHL1 were aberrantly downregulated in COPD, while miR-409-3p was upregulated.
2. Overexpression of SNHG4 mitigated CSE-mediated inflammation, apoptosis, and oxidative stress in 16HBE cells.
3. The salutary effects of SNHG4 overexpression in 16HBE cells were reversed upon overexpression of miR-409-3p or silencing of FHL1.
4. SNHG4 competitively bound miR-409-3p to modulate FHL1 expression.
5. Through the miR-409-3p/FHL1 axis, SNHG4 inhibited the activation of the MAPK signaling pathway in the context of COPD.

**Supplementary Information:**

The online version contains supplementary material available at 10.1186/s40001-024-01872-x.

## Introduction

Chronic obstructive pulmonary disease (COPD) is a prevalent chronic inflammatory disorder of the airways, characterized by progressive airway inflammation and a relentless decline in lung function [1]. The increasing prevalence and mortality associated with COPD pose substantial threats to global health security and economic stability [2]. The etiology of COPD is multifactorial, encompassing exposure to cigarette smoke (CS), second-hand smoke, occupational pollutants, advancing age, and genetic predispositions, with CS identified as the predominant risk factor [3, 4]. While smoking cessation is deemed the most effective therapeutic intervention for individuals with COPD, it is associated with increased anxiety and depression in quitting individuals [5]. To date, the development of specific and efficacious therapeutic strategies for COPD remains elusive, accentuating the imperative for identifying reliable biomarkers and unraveling the disease’s pathogenetic mechanisms.

Long non-coding RNAs (lncRNAs) are identified as crucial regulators exceeding 200 nucleotides in length, playing pivotal roles in various biological pathways [6]. Emerging research has implicated the dysregulated expression of lncRNAs in the pathogenesis of COPD, actively involving them in cellular proliferation, apoptosis, inflammatory response, and airway remodeling [5]. Notably, lncRNA Small Nucleolar RNA Host Gene 5 is reported to be downregulated in COPD, attenuating associated cellular proliferation, apoptosis, and inflammation [7]. Similarly, lncRNA nicotinamide nucleotide transhydrogenase antisense RNA 1 has been highlighted to modulate the pathological aspects of COPD through the microRNA-582-5p/F-box protein 11 axis [8]. LncRNA Small Nucleolar RNA Host Gene 4 (SNHG4) emerges as a novel lncRNA associated with a myriad of human pathologies, including various cancers [9, 10], neonatal pneumonia [11], and cerebral ischemia–reperfusion injury [12]. Furthermore, SNHG4 has been demonstrated to exert anti-inflammatory effects, notably inhibiting M1 polarization of microglia, reducing the production of inflammatory cytokines such as interleukin-6 (IL-6) and tumor necrosis factor-alpha (TNF-α), and blocking the activation of the nuclear factor kappa-light-chain-enhancer of activated B cells (NF-κB) pathway [12]. Recent investigations have corroborated SNHG4’s role in attenuating lipopolysaccharide (LPS)-induced pulmonary inflammation [11]. However, the precise biological functions and mechanistic pathways of SNHG4 in the context of COPD remain to be elucidated, underscoring a gap in our comprehensive understanding of COPD pathogenesis and a potential avenue for therapeutic intervention.

Recently, the competing endogenous RNA (ceRNA) hypothesis has garnered widespread attention for its critical role in COPD [13]. This hypothesis posits that lncRNAs function as ceRNAs, competitively binding miRNAs and thus preventing the miRNA-mediated degradation of target messenger RNAs (mRNAs) [8]. In our predictive analysis, we identified miRNA associated with SNHG4, specifically miR-409-3p, and its target gene, Four and a Half LIM Domains 1 (FHL1). MiR-409-3p, characterized as a short non-coding RNA, is known for its role in downregulating gene expression and modulating a myriad of biological processes, including proliferation, apoptosis, and inflammation. Previous studies have documented the upregulation of miR-409-3p in pediatric patients suffering from adenovirus-induced pneumonia [14]. Moreover, miR-409-3p has been implicated in the regulation of apoptosis and inflammatory damage in WI-38 cells induced by LPS [15]. FHL1 has been previously reported to be associated with Cigarette Smoke Extract (CSE)-induced COPD, exhibiting downregulation in the disease state [16]. Therefore, we hypothesize that SNHG4 may modulate the progression of COPD through targeting the miR-409-3p/FHL1 axis.

In this study, we hypothesize that SNHG4 may exert a protective role in COPD. We investigated the expression patterns of SNHG4 in patients with COPD and explored its biological function in the disease through both in vivo and in vitro experiments. Additionally, we examined the downstream molecular mechanisms of SNHG4 through functional rescue experiments, providing insights into its potential regulatory impact on COPD progression.

## Materials and methods

### Clinical tissue specimens

Forty non-smoking patients with COPD, 40 smoking patients with COPD, and 40 non-smoking, non-COPD subjects were recruited from the Sixth Medical Center of PLA General Hospital. COPD patients were diagnosed using pulmonary function tests and computed tomography scans. All clinical blood and lung tissue samples were preserved at − 80 °C for subsequent analysis. Pulmonary function testing was conducted by a trained physician at our institution. This study received approval from the Sixth Medical Center of PLA General Hospital (No. CH20170306654) Review Board and informed consent was obtained from all participants. Demographic and clinical data are presented in Table 1.

**Table 1:** Clinical characteristics

Characteristic	Non-smokers (*n* = 40)	Smokers (*n* = 40)	COPD (*n* = 40)
Gender			
Male	22	28	26
Female	18	12	14
Age (years)	65.9 ± 6.8	67.5 ± 6.6	66.7 ± 7.2
Hypertension (%)	26.5	38.4	41.2
Diabetes mellitus (%)	17.2	22.6	21.2
Smoking duration (years)	0	29.2 ± 4.2	32.5 ± 5.7
COPD history			
Yes	–	–	12
No	–	–	28
BMI (kg/m^2^)	20.5 ± 1.2	24.1 ± 2.2	25.5 ± 1.8
FEV1 (L)	3.4 ± 0.7	2.6 ± 0.3	2.0 ± 0.4
FEV1%	95.2 ± 4.5	67.3 ± 5.8	44.7 ± 5.2
FEV1/FVC ratio	80.5 ± 6.4	73.8 ± 4.5	55.3 ± 4.7
IL-1β	3.3 ± 1.0	6.2 ± 1.8	13.2 ± 3.2
IL-6	9.5 ± 3.2	16.3 ± 5.5	23.5 ± 6.5
TNF-α	10.5 ± 5.7	14.2 ± 4.5	18.5 ± 6.8

*COPD* chronic obstructive pulmonary disease, *BMI* body mass index, *FEV1* forced expiratory volume in the first second, *FEV1%* forced expiratory volume in the first second as a percentage of the predicted value, *FVC* forced vital capacity, *IL-1β* interleukin 1 beta, *IL-6* interleukin 6, *TNF-α* tumor necrosis factor alpha

### Cell culture and treatment

Bronchial epithelial cells treated with CSE are widely utilized in vitro models for COPD. 16HBE cells, purchased from the American Type Culture Collection (ATCC, USA), were cultured at 37 °C and 5% CO_2_ in Roswell Park Memorial Institute-1640 medium (RPMI-1640) supplemented with 10% fetal bovine serum (FBS, Invitrogen). Different concentrations (1%, 2%, 3%, and 4%) of CSE were prepared using ten cigarettes. The smoke from ten cigarettes was bubbled through 25 mL of medium, the suspension was titrated to pH 7.4, filter sterilized, and considered as 100% CSE. CSE samples were diluted with phosphate buffered saline (PBS) to concentrations of 1%, 2%, 3%, and 4% and stored at − 80 °C. 16HBE cells were treated with CSE for 24 h, while control cells were treated with a similar dose of PBS [13].

### Cell transfection

Overexpression plasmid vectors and short hairpin RNAs (shRNAs) targeting SNHG4 and FHL1, along with negative controls (pcDNA 3.1-SNHG4/FHL1, sh-SNHG4/FHL1, pcDNA 3.1, sh-NC), miR-409-3p mimics/inhibitors, and mimic/inhibitor-NCs were purchased from GenePharma (Shanghai, China). Post-CSE treatment, the oligonucleotides or plasmid vectors were transfected into cells using Lipofectamine 2000 transfection reagent (Invitrogen, USA). Transfection efficiency was assessed 48 h later via quantitative reverse transcription polymerase chain reaction (RT-qPCR) or Western blot. To mitigate off-target effects, three distinct shRNAs targeting FHL1 were designed (see Additional file 2: Table S1 for sequence information), and the most efficacious shRNA was selected for subsequent experiments. To inhibit the Mitogen-Activated Protein Kinase (MAPK) pathway, cells were treated with a p38 inhibitor (SB203580, Alexis Corporation) [17].

### RT-qPCR

Expression levels of SNHG4, miR-409-3p, and FHL1 were evaluated using RT-qPCR. Total RNA was extracted from lung tissues and cells using Trizol reagent (Invitrogen, USA). Reverse transcription of LncRNA/mRNA and miRNA was conducted using lnRcute lncRNA cDNA kit (TIANGEN, China) and Rcute Plus miRNA cDNA kit (TIANGEN, China), respectively. RT-qPCR was performed using SYBR Green reagent kits (Thermo Fisher Scientific, Waltham, MA, USA) and the Mx3005P QPCR system (Agilent Technologies, Santa Clara, CA, USA). U6 served as the internal control gene for mRNA. Glyceraldehyde 3-phosphate dehydrogenase (GAPDH) served as the internal reference gene for mRNA and lncRNA. Primer sequences are available in Table 2. All experiments were conducted with three biological replicates and three technical replicates [18].

**Table 2:** Primer sequences

	Primer sequences (5′–3′)
SNHG4	Forward: 5′-GGCTAGAGTACAGTGGCTCG-3′
	Reverse: 5′-GCAAATCGCAAGGTCAGG-3′
miR-409-3p	Forward: 5′-GCCGAGGAATGTTGCTCGGTG-3′
	Reverse: 5′-CTCAACTGGTGTCGTGGA-3′
FHL1	Forward: 5′-CTGGGTTTGGTAAAGGCTCC-3′
	Reverse: 5′-GGCACAGTCGGGACAATACAC-3′
U6	Forward: 5′-CTCGCTTCGGCAGCACA-3′
	Reverse: 5′-AACGCTTCACGAATTTGCGT-3′
GAPDH	Forward: 5′-TCCCATCACCATCTTCCA-3′
	Reverse: 5′-CATCACGCCACAGTTTTCC-3′

*SNHG4* long non-coding RNA small nucleolar host gene 4, *miR-409-3p* microRNA-409-3p, *FHL1* Four-and-a-Half LIM Domains 1, *GAPDH* glyceraldehyde-3-phosphate dehydrogenase

## Western blot

Total proteins from cells and tissues were extracted using 500 µL of Radio-Immunoprecipitation Assay lysis buffer (Beyotime, China). Equal amounts of protein (20 µg) were loaded onto 8% sodium dodecyl sulfate–polyacrylamide gel electrophoresis (SDS-PAGE, Solarbio) and transferred onto polyvinylidene difluoride (PVDF) membranes (Invitrogen). The membranes were blocked with 5% skim milk for 1 h and then incubated with primary antibodies at 4 °C overnight. Horseradish peroxidase-conjugated goat anti-rabbit secondary antibodies IgG (1:1000, ab181236, Abcam) were added and incubated for 2 h. Signals were visualized using the Enhanced Chemiluminescence (ECL) reagent kit (34080, Thermo Fisher Scientific, Waltham, Massachusetts, USA). Density analysis was performed using ImageJ software. Commercial antibodies used in the experiment included: FHL1, B-cell lymphoma-2 (Bcl-2), Bcl-2-associated X protein (Bax), cleaved-caspase-3, mitogen-activated protein kinase kinase 1 (MEK1), phosphorylated-MEK1 (p-MEK1), p38 MAPK, phosphorylated-p38 MAPK (p-p38 MAPK), and GAPDH (GAPDH and FHL1 from Abcam; others from Cell Signaling Technology, dilution for FHL1 was 1:500, and for others 1:1000) [19].

### Biochemical marker detection

Levels of factors associated with airway remodeling and inflammatory response, including alpha-smooth muscle actin (α-SMA), collagen I, IL-1β, IL-6 and TNF-α, were measured in patient blood, mouse lung tissue, and cell supernatant using enzyme-linked immunosorbent assay (ELISA) kits (Thermo Fisher Scientific, Waltham, Ma, USA) according to the manufacturer’s instructions. Absorbance at 450 nm was assessed using a Centro LB 960 microplate reader (BERTHOLD, Stuttgart, Germany). Malondialdehyde (MDA) and superoxide dismutase (SOD) levels were detected using standard kits (Nanjing Jiancheng Bioengineering Institute, China) [20].

### Cell counting kit-8 (CCK-8)

Post-transfection, 16HBE cells (1 × 10^6^ cells/well) were seeded in 6-well plates and treated with 10 µL of CCK-8 reagent (cat. no. 96992-100TESTS-F; Sigma-Aldrich; Merck KGaA) at different time points (0, 24, 48, and 72 h) and incubated at 37 °C for 2 h. The optical density (OD) at 450 nm was recorded using a Multiskan microplate reader (Thermo Fisher Scientific, Inc.) [21].

### 5-Ethynyl-2′-deoxyuridine (EdU) assay

Treated 16HBE cells (5 × 10^4^ cells/well) were seeded on a 96-well plate and incubated in EdU medium for 2 h. Cells were washed with PBS and fixed with 4% paraformaldehyde. The EdU assay was conducted using an EdU detection kit (RiboBio, Guangzhou, China). Cell nuclei were stained with 4′,6-diamidino-2-phenylindole (DAPI) solution, and Edu positive rates were observed under a fluorescence microscope (Nikon-OLYMPUS IX71, Nikon Instruments, Japan) [22].

### Flow cytometry

Cell apoptosis was detected using the Annexin V-FITC/propidium iodide (PI) Apoptosis Detection Kit (Sangon Biotech Co., Ltd). Treated 16HBE cells were resuspended in binding buffer (300 µL). Subsequently, cells were dual-stained with 5 μL Annexin V-FITC and 5 μL PI for 20 min in dark, room temperature conditions. Finally, the percentage of apoptotic cells was evaluated using a FACS Calibur flow cytometer [20]. Quadrant definitions: Q1 (upper left quadrant) is not typically used for traditional Annexin V/PI apoptosis assays; Q2 (upper right quadrant) usually represents late apoptotic or dead cells; Q3 (lower left quadrant) usually indicates live cells; Q4 (lower right quadrant) typically represents early apoptotic cells.

### Dual-luciferase reporter assay

16HBE cells were plated in 24-well plates. Constructs containing wild type or mutant binding sites of SNHG4 or FHL1 3′ untranslated region (UTR) (Promega) were inserted into pmirGLO vector (Promega, Madison, WI), named SNHG4/FHL1-WT 3′UTR and SNHG4/FHL1-MUT 3′UTR reporter genes, respectively. The respective luciferase reporter genes were co-transfected with miR-409-3p mimic or miR-NC into 16HBE cells. After 48 h of incubation, luciferase activity was measured using a Dual-Luciferase Reporter Assay Kit (Promega) [23].

### RNA immunoprecipitation (RIP) assay

The assay was conducted using anti-Ago2 (ab252812) and anti-IgG (ab109489) antibodies. In brief, 16HBE cells were lysed, and then incubated with protein-G magnetic beads conjugated with anti-Ago2 or IgG antibodies at 40 °C for 6 h. Beads were collected, bound RNA was isolated, and the enrichment levels of SNHG4, miR-409-3p, and FHL1 were detected [13].

### Animal experiments

Animal studies were authorized by the Animal Care and Use Committee of the Sixth Medical Center of PLA General Hospital (No. 201803CH6) and strictly followed the animal research charter. Twenty-four male C57BL/6 mice (6–8 weeks old) were acquired from Hunan SJA Laboratory Animal Co., Ltd and housed in standard laboratory conditions (12 h light/dark cycle, temperature 24 ± 2 °C, humidity 50%). After a week of acclimatization, mice were randomly divided into four groups: control, CS, sh-SNHG4, and sh-NC. Except for the Control group, remaining mice were exposed to CS from 10 cigarettes using a smoking machine (TE-10, Teague Enterprises) twice daily [24], 5 days a week, for 12 weeks. The total particulate matter concentration measured indoors was 160–180 mg/m^3^. Control group mice were maintained in ambient air. To knock down SNHG4, after the first CS exposure, mice were injected intravenously with a lentiviral vector targeting SNHG4 shRNA (GenePharma, Shanghai, China). After 12 weeks, mice were euthanized, and a portion of lung tissue was fixed in 4% paraformaldehyde for histopathological analysis, while the remainder was preserved at − 80 °C for subsequent gene extraction. Twelve weeks post-treatment, mice were euthanized, and the upper lobe of the left lung was fixed in 4% paraformaldehyde for histopathological analysis. The upper lobe of the right lung was preserved at − 80 °C for subsequent genetic material extraction.

### Pulmonary function testing

Pulmonary function was assessed in mice using a whole-body plethysmograph (Buxco Electronics, Ltd., USA). Briefly, mice were placed randomly in a chamber connected to a sensitive pressure transducer, which measures slight pressure changes inside the chamber. The expiration time (Te), relaxation time (Tr), peak inspiratory flow (PIF), and peak expiratory flow (PEF) are all parameters reflecting restricted airflow. Enhanced pause (Penh, Penh = (Te/Tr − 1) × (PEF/PIF)) was recorded using FinePoint software (Buxco Electronics, Ltd., USA) when mice were quiet to evaluate pulmonary resistance. Values were averaged and reported as absolute Penh values.

### Hematoxylin and eosin (H&E) staining

Lung tissue samples were fixed in 4% paraformaldehyde, embedded in paraffin, then cut into consecutive 4-μm-thick sections and stained with H&E using widely adopted standard procedures. Sections were observed under a microscope (Nikon, Japan) [25].

### Terminal deoxynucleotidyl transferase dUTP nick end labeling (TUNEL) staining

Apoptotic cells in lung tissues were detected using previous methodologies. Briefly, lung tissue sections were deparaffinized and rehydrated. They were then treated with proteinase K (20 ng/μL). Sections were incubated with bromodeoxyuridine (BrdU) solution at 37 °C for 1 h, then incubated with BrdU antibody, followed by three 5-min washes in PBS. All sections were counterstained with DAPI (20 mmol/L) at room temperature in the dark. Images were captured using a fluorescence microscope (Leica, Germany), and TUNEL-positive cells were manually counted in each microscopic image [26].

### Immunohistochemistry (IHC) analysis

FHL1 expression was assessed through IHC [27]. Briefly, deparaffinized and rehydrated sections were blocked with 3% hydrogen peroxide. Tissue sections were incubated with FHL1 antibody (ab23937, Abcam) overnight at 4 °C. Subsequently, sections were incubated with biotinylated anti-rabbit secondary antibody (1:250) at room temperature for 1 h. Finally, sections were incubated with ABC reagent kit provided peroxidase substrate solution and developed with diaminobenzidine. Sections were counterstained with hematoxylin. Images were taken using an optical microscope (Olympus, Japan).

### Statistical analysis

Data were analyzed using SPSS 21.0 (SPSS, Inc, Chicago, IL, USA) statistical software. The data were confirmed to be normally distributed by the Kolmogorov–Smirnov test. Results are expressed as mean ± standard deviation (SD). Comparisons between two groups were made using *t*-tests, and comparisons among multiple groups were made using one-way analysis of variance (ANOVA). Two-way analysis of ANOVA was employed to evaluate the variations in inflammatory markers, SNHG4, and FEV1% among patients with COPD, non-smokers, and smokers. *P*-value of < 0.05 was considered statistically significant. All experiments were performed with at least three biological replicates.

## Results

### SNHG4 manifests a distinct downregulation in the context of COPD and is inversely correlated with inflammatory biomarkers

**Fig. 1:**
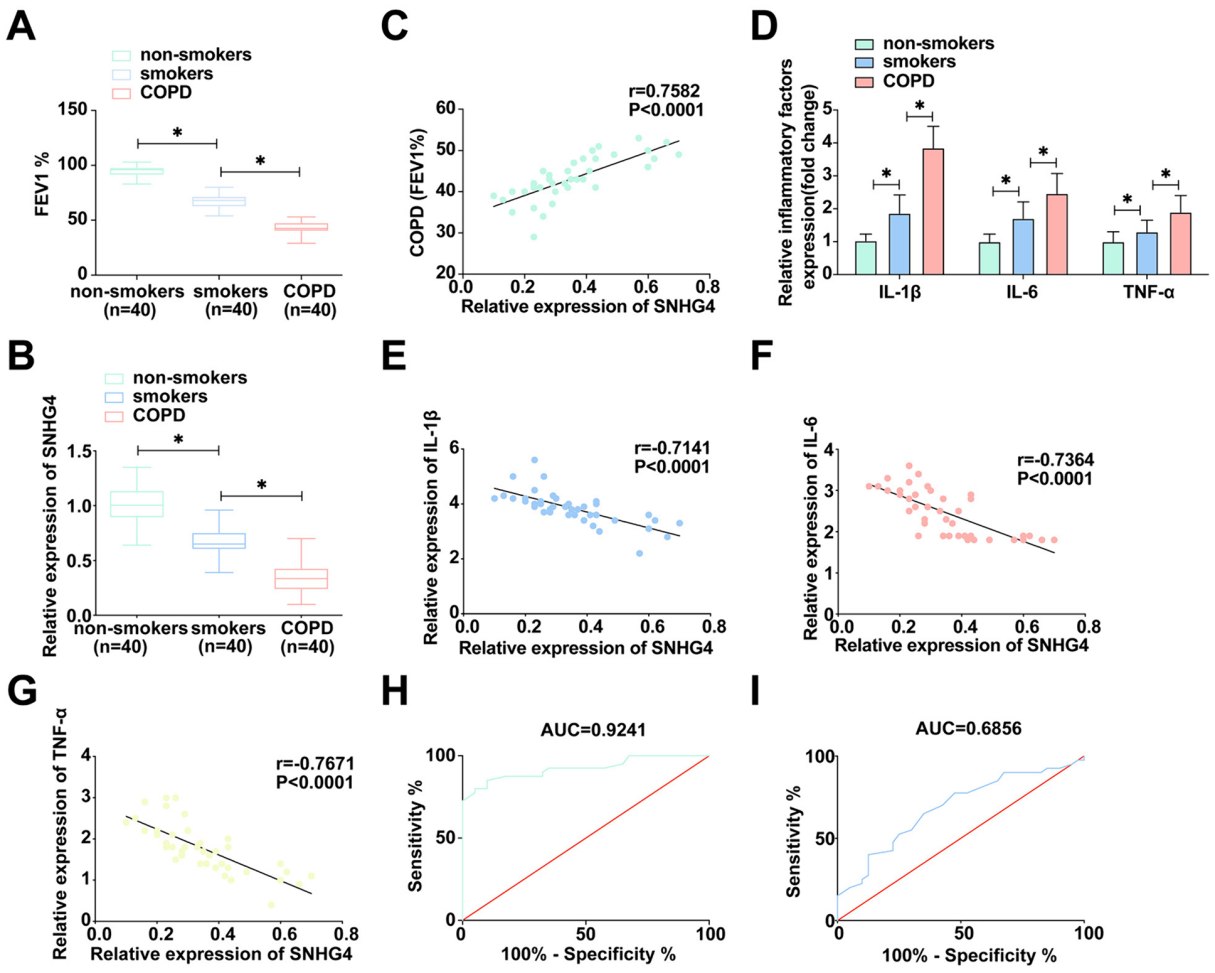
Regulation of SNHG4 in COPD and its correlation with inflammation. **A** Pulmonary function tests measure FEV1% levels in clinical lung specimens. **B** The expression of SNHG4 in clinical lung samples is quantified. **C** Pearson correlation analysis assesses the relationship between SNHG4 and FEV1%. **D** ELISA quantifies the expression of IL-1β, IL-6, and TNF-α in clinical lung samples. **E**–**G** Pearson correlation analysis evaluates the correlations between SNHG4 and IL-1β, IL-6, TNF-α, respectively. **H** ROC analysis of SNHG4 in differentiating COPD patients from non-smokers. **I** ROC analysis of SNHG4 in distinguishing COPD patients from smokers. Data are presented as mean ± SD (*n* = 40). *P < 0.05

To elucidate SNHG4’s involvement in COPD, a study cohort including smokers, non-smokers, and individuals diagnosed with COPD was established to analyze pulmonary function. As delineated in Fig. 1A, FEV1% is significantly compromised in both smokers and COPD patients relative to non-smokers, with the most severe reduction observed in COPD sufferers. Further, RT-qPCR analyses confirmed a marked decrease in SNHG4 expression within COPD patients as shown in Fig. 1B. Clinical correlation analysis yielded a positive relationship between SNHG4 expression and FEV1% in the COPD demographic (Fig. 1C). Recognizing the critical involvement of inflammatory cytokines such as IL-1β, IL-6, and TNF-α in COPD pathology [28], their concentrations were measured via ELISA. The results, presented in Fig. 1D and Table 1, indicate significant elevations of these cytokines in smokers and COPD patients compared to non-smokers. Moreover, an inverse correlation was discerned between SNHG4 expression and the levels of IL-1β, IL-6, and TNF-α in COPD patients (Fig. 1E–G). ROC curve analyses further ascertained SNHG4’s efficacy in distinguishing COPD patients from smokers and non-smokers, revealing its potential as a diagnostic biomarker with notable sensitivity and specificity as depicted in Fig. 1H, I. These findings collectively underscore the significant relationship of SNHG4 with the pathogenesis and progression of COPD.

### SNHG4 alleviates CSE-induced pathological alterations in alveolar epithelial cells

**Fig. 2:**
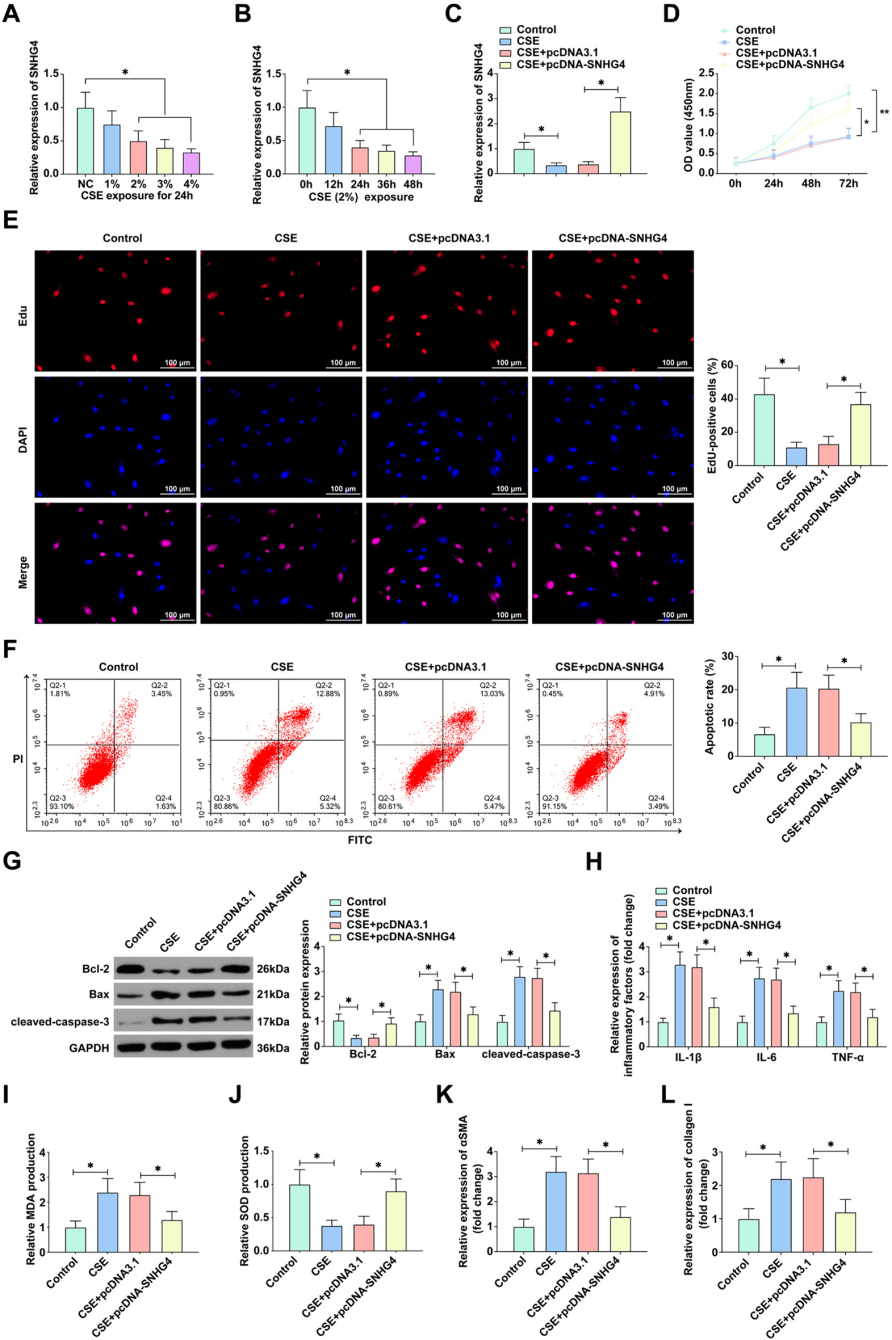
SNHG4 mitigates CSE-induced effects on 16HBE Cell proliferation, apoptosis, inflammation, and airway remodeling. **A** RT-qPCR assesses SNHG4 expression in 16HBE cells exposed to various concentrations of CSE (1%, 2%, 3%, and 4%) for 24 h. **B** RT-qPCR measures SNHG4 expression at different time points under 2.5% CSE exposure. **C** RT-qPCR evaluates the impact of transfection with pcDNA 3.1-SNHG4 on SNHG4 expression in 16HBE cells. **D**, **E** Cell proliferation assessed by CCK-8 and EdU assays. **F** Flow cytometry detects cell apoptosis. **G** Western blot analyzes the levels of apoptosis-related proteins (Bcl-2, Bax, and cleaved-caspase-3). **H** ELISA measures the levels of inflammatory cytokines (IL-1β, IL-6, and TNF-α). **I**, **J** Oxidative stress response evaluated by MDA assay and SOD activity. **K**, **L** ELISA quantifies airway remodeling markers (α-SMA and collagen I). Data are presented as mean ± SD (*n* = 3). *P < 0.05

To dissect the therapeutic potential of SNHG4 in COPD, we employed a CSE-induced 16HBE cellular model, simulating the pathological environment of COPD. Quantitative RT-PCR revealed a dose-dependent attenuation of SNHG4 expression in response to increasing concentrations of CSE, pinpointing a significant reduction particularly at 2% concentration (Fig. 2A). This concentration was selected for further experimentation due to its optimal balance between effectivity and cellular viability. Enhancing SNHG4 levels via transfection with pcDNA 3.1-SNHG4 into CSE-exposed 16HBE cells demonstrated a significant rescue effect. Both CCK-8 and EdU assays indicated that while CSE notably impaired cellular proliferation, SNHG4 overexpression significantly reinstated the proliferative capacity of these cells (Fig. 2D, E). Apoptotic rates, as assessed by flow cytometry, were elevated in CSE-treated cells, a trend that was notably reversed with increased SNHG4 expression, reducing the apoptotic rates back to near-baseline levels (Fig. 2F). Moreover, SNHG4 overexpression mitigated the adverse modulation of apoptosis-related proteins induced by CSE, as evidenced by normalized Bax, cleaved-caspase-3, and Bcl-2 protein levels (Fig. 2G). Given the pivotal role of inflammatory cytokines in COPD pathogenesis, ELISA was employed to assess the impact of SNHG4 on CSE-induced inflammation. Overexpressing SNHG4 attenuated the upregulation of IL-1β, IL-6, and TNF-α, suggesting a dampening effect on the inflammatory milieu provoked by CSE (Fig. 2H). Oxidative stress markers, specifically the elevated MDA and decreased SOD triggered by CSE, were also significantly reversed in cells overexpressing SNHG4, indicating an antioxidative role of SNHG4 (Fig. 2I, J). Lastly, SNHG4 overexpression was found to reduce the levels of α-SMA and collagen I, markers indicative of airway remodeling, suggesting its potential in ameliorating the structural alterations associated with COPD (Fig. 2K, L). Collectively, these findings advocate for SNHG4’s multifaceted role in moderating CSE-induced apoptosis, inflammation, oxidative damage, and promoting cell proliferation and airway remodeling in COPD.

### SNHG4: deciphering its role as a miR-409-3p sponge in COPD

**Fig. 3:**
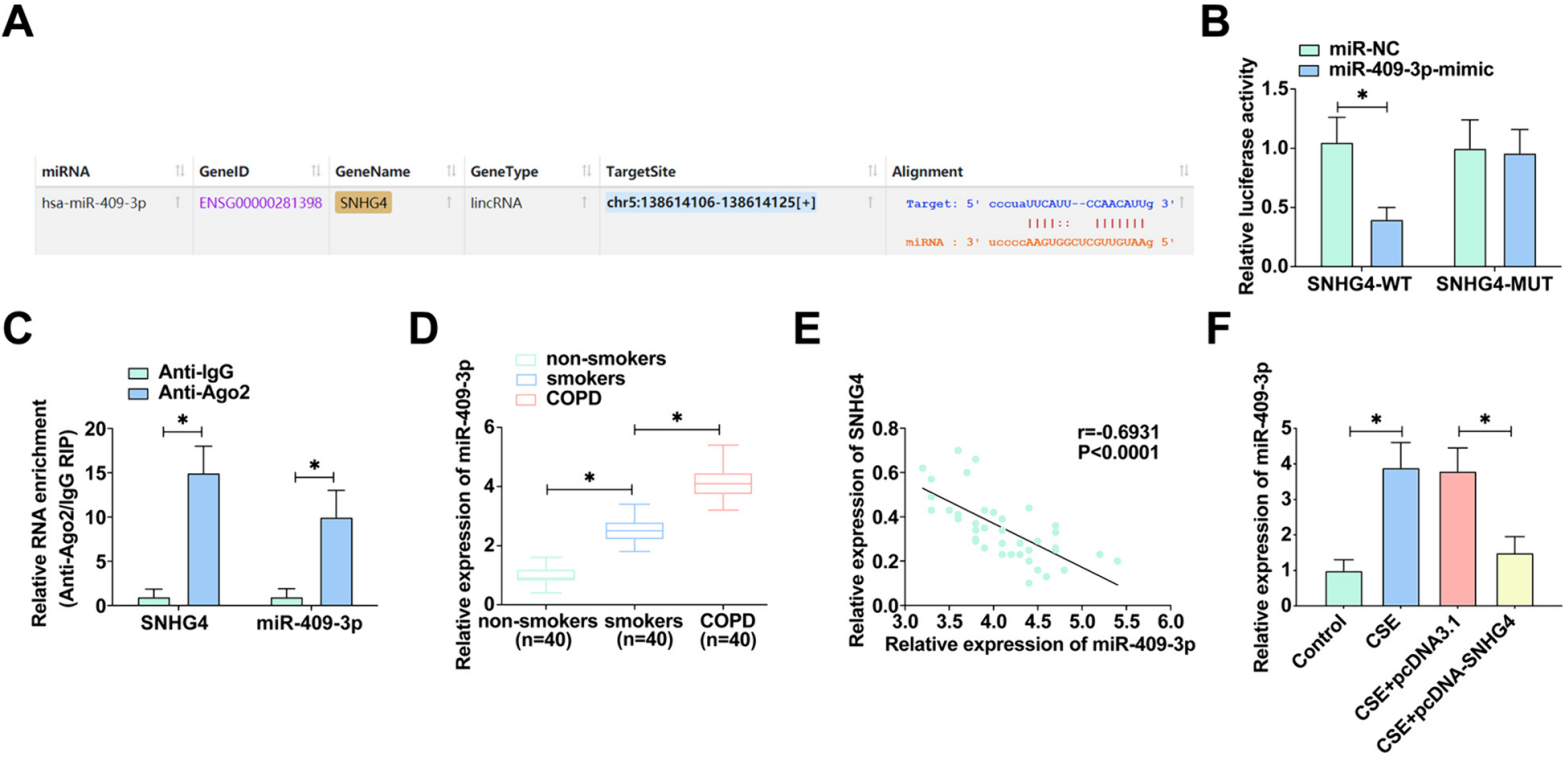
SNHG4 acts as a sponge for miR-409-3p. **A** The binding sites between SNHG4 and miR-409-3p predicted by the bioinformatics website https://starbase.sysu.edu.cn. **B**, **C** The interaction between SNHG4 and miR-409-3p assessed by dual-luciferase reporter assay and RIP experiment. **D** RT-qPCR analyzes miR-409-3p expression in various tissues. **E** Pearson correlation analysis evaluates the correlation between miR-409-3p and SNHG4. **F** RT-qPCR measures miR-409-3p expression in 16HBE cells under different treatments. Data are presented as mean ± SD (*n* = 3). *P < 0.05

Investigating the sophisticated regulatory network of COPD, we centered our focus on SNHG4’s interaction with miRNAs. Bioinformatics predictions from starBase [29] identified a potential interaction between SNHG4 and miR-409-3p (Fig. 3A). To confirm this, a dual-luciferase reporter assay was employed, demonstrating a marked repression of luciferase activity of SNHG4-WT 3′UTR by miR-409-3p mimic, whereas its mutant counterpart showed no such suppression (Fig. 3B). This specificity of interaction accentuates SNHG4’s function as a molecular sponge for miR-409-3p. Further corroboration was achieved through RIP assays, which revealed significant co-enrichment of miR-409-3p and SNHG4 in RNA-induced silencing complexes associated with Ago2, underscoring the miRNA-mediated post-transcriptional regulation (Fig. 3C). Additionally, miR-409-3p was found to be abundantly expressed in COPD tissues (Fig. 3D), and clinical correlation analysis depicted an inverse relationship between miR-409-3p and SNHG4 levels in COPD patients (Fig. 3E). Notably, in CSE-treated 16HBE cells, miR-409-3p expression was significantly upregulated, while transfection with pcDNA-SNHG4 or miR-409-3p inhibitor successfully mitigated this expression (Fig. 3F). These findings collectively illuminate the regulatory axis of SNHG4 and miR-409-3p in COPD, positioning SNHG4 as a key modulator in the disease’s molecular milieu.

### Regulatory interplay between SNHG4 and miR-409-3p mediates CSE-induced changes in alveolar epithelial cell function

**Fig. 4:**
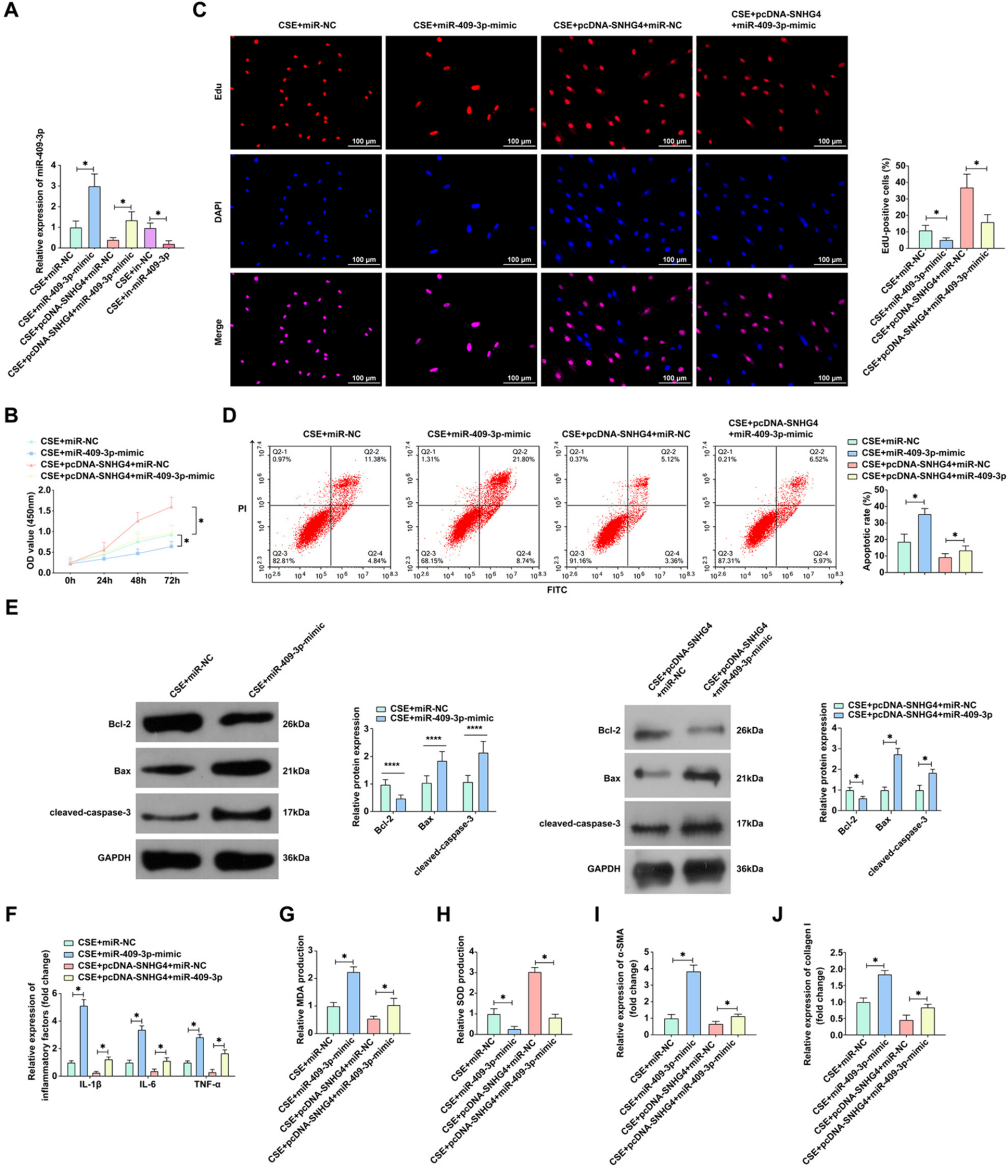
Overexpression of SNHG4 alleviates CSE effects on 16HBE cells by suppressing miR-409-3p expression. **A** RT-qPCR measures miR-409-3p expression in 16HBE cells under various treatments. **B**, **C** Cell proliferation assessed by CCK-8 and EdU assays. **D** Flow cytometry detects cell apoptosis. **E** Western blot analyzes expression of apoptosis-related proteins (Bcl-2, Bax, and cleaved-caspase-3). **F** ELISA measures levels of inflammatory cytokines (IL-1β, IL-6, and TNF-α). **G**, **H** Oxidative stress response evaluated by ROS assay and SOD activity. **I**, **J** ELISA quantifies airway remodeling markers (α-SMA and collagen I). Data are presented as mean ± SD (*n* = 3). *P < 0.05

To explore the regulatory crosstalk between SNHG4 and miR-409-3p in modulating cellular responses to CSE in alveolar epithelial cells, we co-transfected 16HBE cells with pcDNA-SNHG4 and miR-409-3p mimic following CSE treatment. RT-qPCR results indicated that while miR-409-3p mimic augmented miR-409-3p expression, pcDNA-SNHG4 counteracted this effect, highlighting the reciprocal regulatory relationship between SNHG4 and miR-409-3p (Fig. 4A). In an illustrative series of experiments (Fig. 4B–E), pcDNA-SNHG4 was shown to enhance cell viability and suppress apoptotic activity in CSE-treated cells, effects which were negated upon introduction of miR-409-3p mimic, underscoring the potent modulatory role of SNHG4 overexpression. Further analysis demonstrated that the miR-409-3p mimic could reverse the mitigative effects of SNHG4 overexpression on CSE-induced inflammation and oxidative damage, pointing to a critical nexus of regulation and control mediated by SNHG4 and miR-409-3p (Fig. 4F–H). Most crucially, the miR-409-3p mimic was seen to overturn the remedial impact of pcDNA-SNHG4 on airway remodeling triggered by CSE exposure (Fig. 4I, J). These findings collectively illuminate the significant role of SNHG4 overexpression in alleviating CSE-induced perturbations in cell proliferation, apoptosis, inflammation, and airway remodeling, primarily through the suppression of miR-409-3p expression. This intricate regulatory axis potently influences the cellular adaptive and pathophysiological responses in COPD, suggesting SNHG4 and miR-409-3p as potential therapeutic targets for ameliorating disease progression.

### Targeted regulation of FHL1 by miR-409-3p

**Fig. 5:**
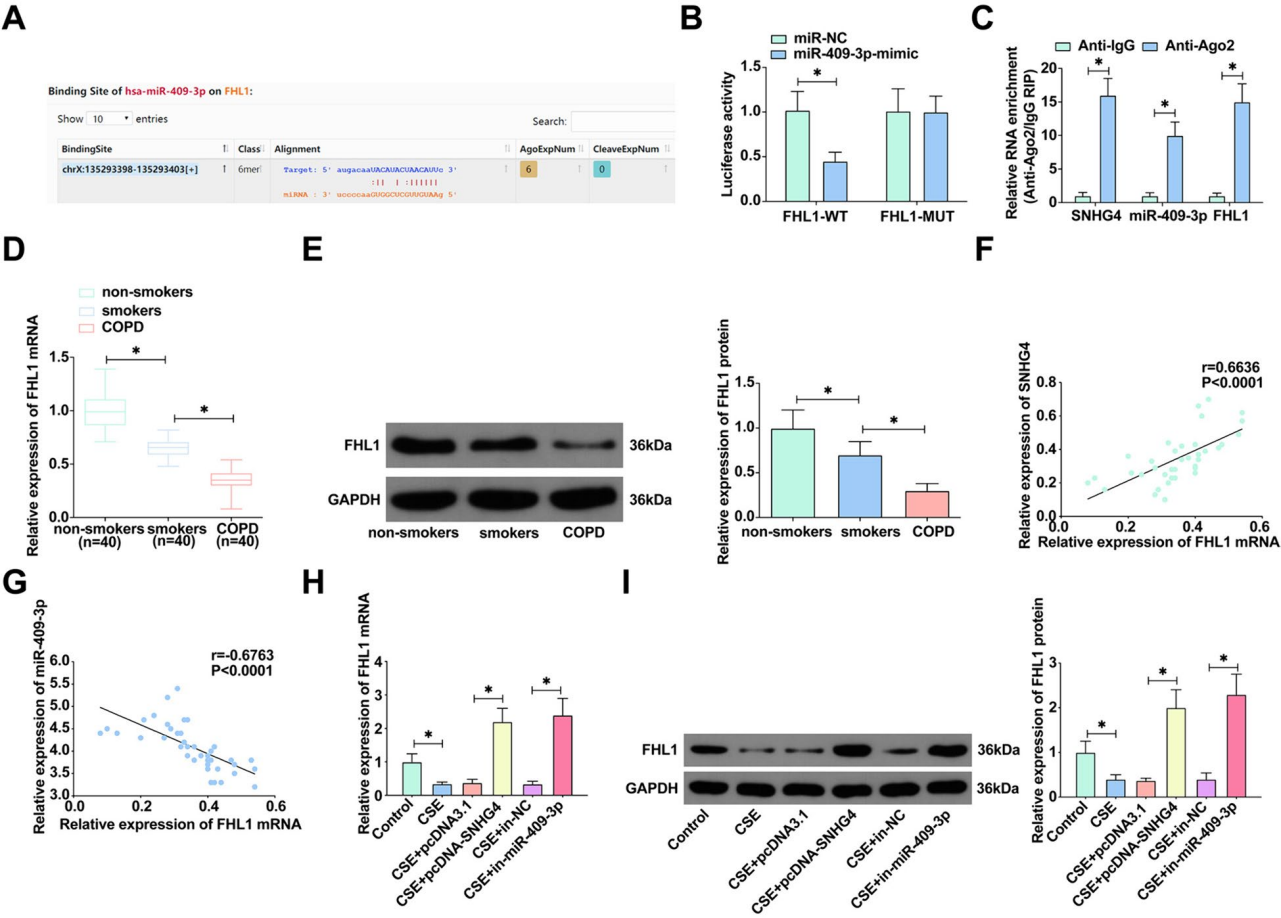
miR-409-3p directly targets FHL1. **A** The binding sites between FHL1 and miR-409-3p predicted by the bioinformatics website https://starbase.sysu.edu.cn. **B**, **C** The interaction between FHL1 and miR-409-3p assessed by dual-luciferase reporter assay and RIP experiment. **D**, **E** RT-qPCR and Western blot analyze FHL1 expression in clinical lung samples. **F**, **G** Pearson correlation analysis evaluates the correlation between FHL1, miR-409-3p, and SNHG4. **H**, **I** RT-qPCR and Western blot measure FHL1 expression in 16HBE cells under various treatments. Data are presented as mean ± SD (*n* = 3). *P < 0.05

Continuing our exploration into the molecular underpinnings of COPD, we delved into the potential downstream targets of miR-409-3p. Predictive interactions identified through the starBase database unveiled a potential binding site between miR-409-3p and FHL1 (Fig. 5A). Dual-luciferase reporter assays corroborated these predictions, revealing a substantial diminution in luciferase activity for FHL1-WT 3′UTR in the presence of the miR-409-3p mimic, whereas such suppression was not observed with the FHL1-MUT 3′UTR variant (Fig. 5B). This specific interaction was further substantiated through RIP assays, showing significant enrichment of miR-409-3p and FHL1 in RNA-induced silencing complexes precipitated with Ago2 antibody (Fig. 5C). Turning our attention to clinical lung samples, we noted a conspicuous downregulation of FHL1 in COPD patients (Fig. 5D, E). Clinical correlation analyses revealed a positive correlation between FHL1 and SNHG4 expression and a negative correlation with miR-409-3p expression in COPD patients (Fig. 5F, G). Furthermore, in CSE-treated 16HBE cells where FHL1 expression was notably compromised, upregulation of SNHG4 or inhibition of miR-409-3p was observed to enhance FHL1 expression (Fig. 5H, I). These comprehensive findings underscore FHL1 as a downstream gene of miR-409-3p, illustrating a critical regulatory axis in the pathophysiology of COPD.

### The role of SNHG4 in ameliorating CSE-induced pathological changes in alveolar epithelial cells via FHL1 regulation

**Fig. 6:**
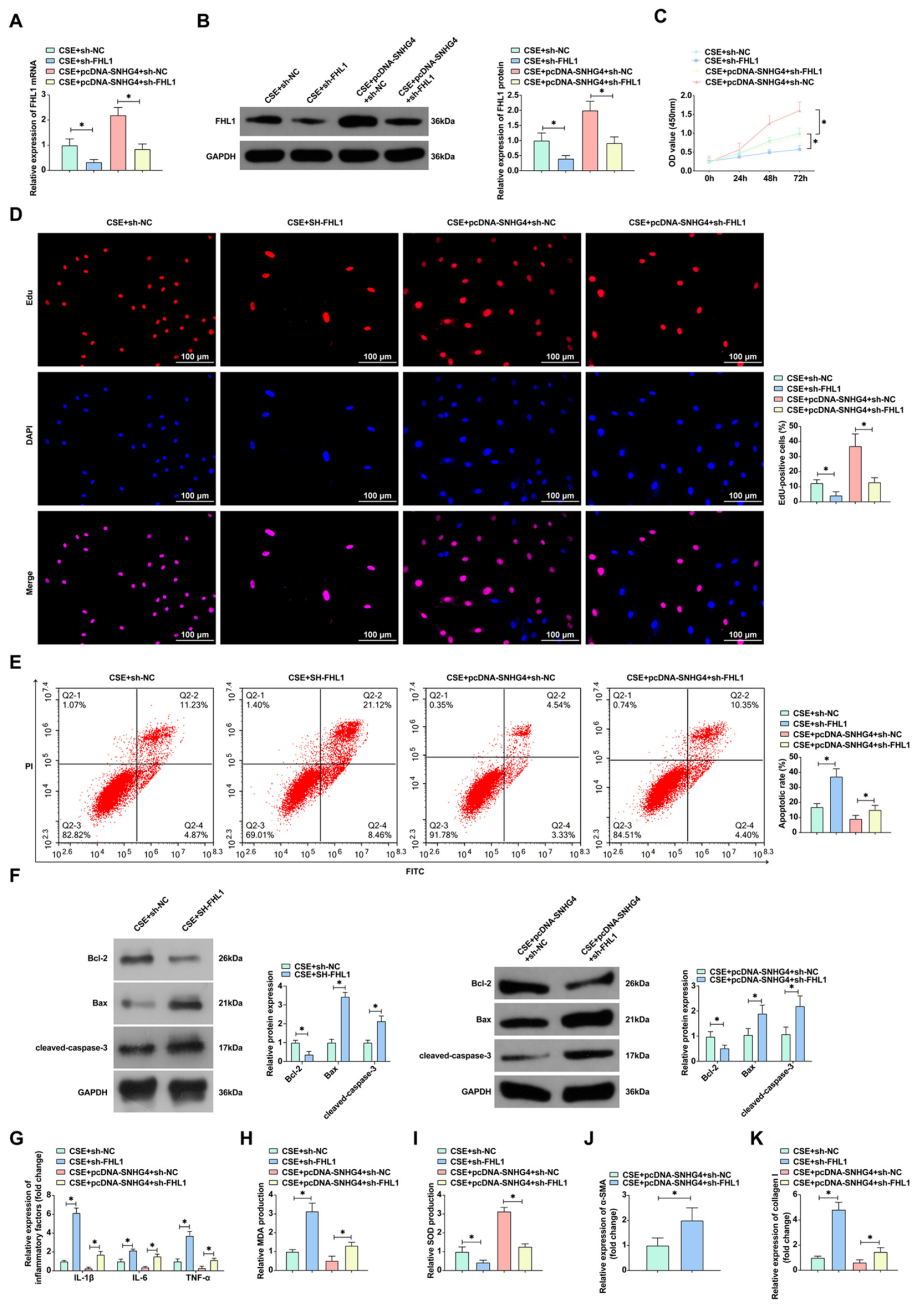
SNHG4 regulates COPD progression via the miR-409-3p/FHL1 axis. **A**, **B** RT-qPCR and Western blot assess the impact of transfection with pcDNA 3.1-SNHG4 and sh-FHL1 on FHL1 mRNA and protein expression. **C**, **D** Cell proliferation evaluated by CCK-8 and EdU assays ( ×60). **E** Flow cytometry measures cell apoptosis. **F** Western blot analyzes the levels of apoptosis-related proteins (Bcl-2, Bax, and cleaved-caspase-3). **G** ELISA quantifies the levels of inflammatory cytokines (IL-1β, IL-6, and TNF-α). **H**, **I** Oxidative stress response assessed by MDA assay and SOD activity. **J**, **K** ELISA measures airway remodeling markers (α-SMA and collagen I). Data are presented as mean ± SD (*n* = 3). *P < 0.05

Investigating SNHG4’s functional impact through the miR-409-3p/FHL1 axis on COPD progression, we embarked on a series of functional rescue experiments. Co-transfection of pcDNA 3.1-SNHG4 and sh-FHL1 into and 16HBE cells treated with CSE provided a platform to dissect the interplay between SNHG4 and FHL1. RT-qPCR and Western blot findings indicated that sh-FHL1 significantly reversed the upregulation of FHL1 instigated by pcDNA-SNHG4, delineating the regulatory influence of SNHG4 on FHL1 expression (Fig. 6A, B). Subsequent proliferation assays, including CCK8 and EdU, showed that knockdown of FHL1 abolished the proliferative enhancement induced by SNHG4 overexpression in CSE-induced 16HBE cells, suggesting a critical role of FHL1 in SNHG4-mediated cell growth (Fig. 6C, D). Additionally, the reduction of apoptosis facilitated by SNHG4 overexpression was counteracted by FHL1 knockdown, as evidenced by increased apoptotic markers in the modified cells (Fig. 6E, F). The mitigation of inflammation and oxidative damage by SNHG4 upregulation was also reversed upon FHL1 knockdown, highlighting the intertwined regulatory effects of these molecules in cellular stress responses (Fig. 6G–I). Notably, in the context of airway remodeling, FHL1 knockdown led to increased levels of α-SMA and collagen I, thus reversing the beneficial effects of SNHG4 upregulation on airway structural changes induced by CSE (Fig. 6J, K). These results collectively illuminate the modulatory capacity of SNHG4 through the miR-409-3p/FHL1 axis in COPD progression, underscoring its potential as a therapeutic target for managing the disease.

### SNHG4 intervention in MAPK pathway activation: deciphering the miR-409-3p/FHL1 axis in COPD

**Fig. 7:**
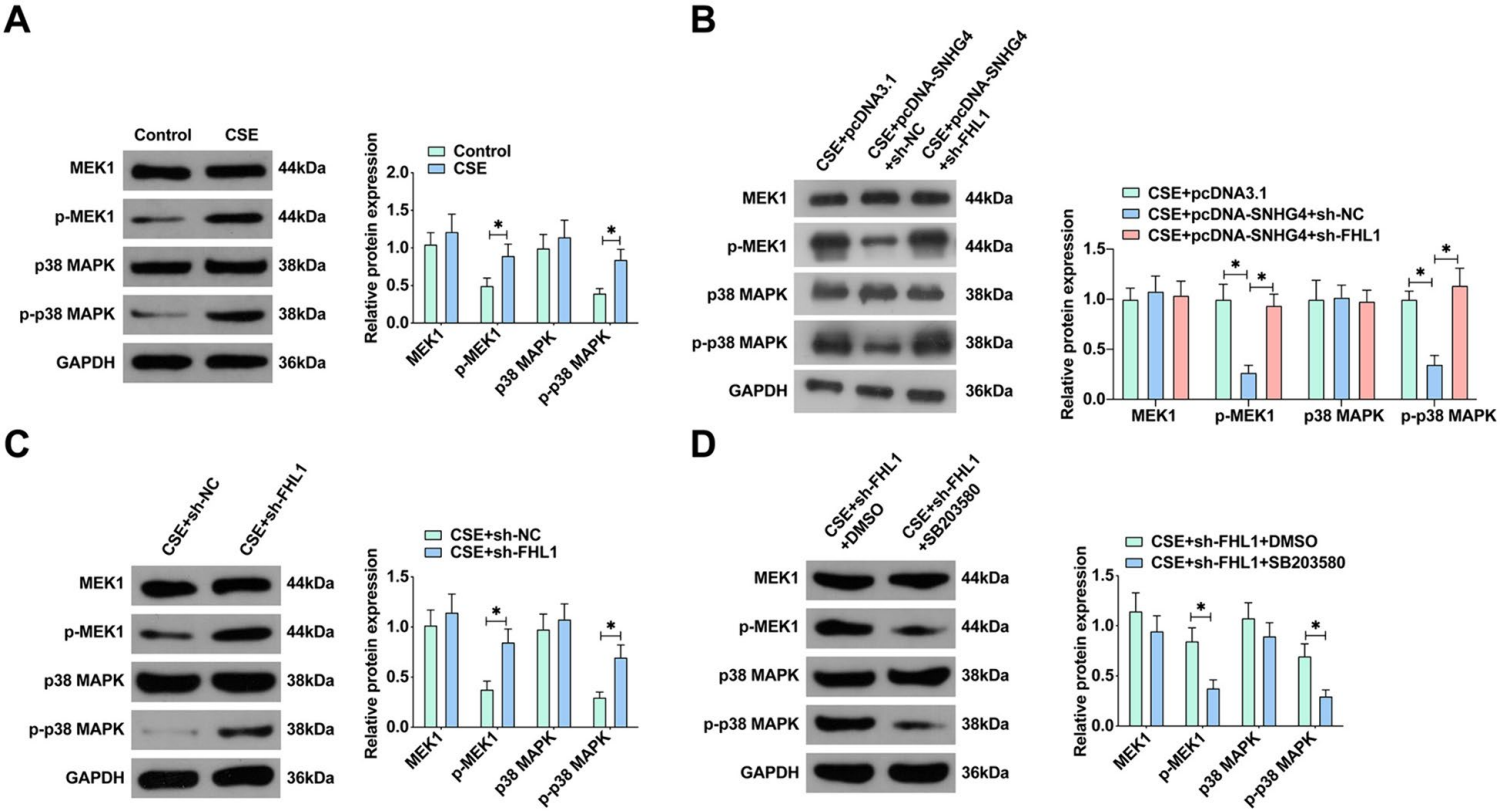
SNHG4 regulates the MAPK signaling pathway via the miR-409-3p/FHL1 axis. **A** Western blot analyzes the expression of MAPK pathway proteins in 16HBE cells treated with CSE. **B** Western blot evaluates the expression of MAPK pathway proteins following co-transfection with pcDNA-SNHG4 and sh-FHL1. **C** Western blot examines the expression of MAPK pathway proteins after FHL1 knockdown. **D** Western blot analysis of MAPK pathway protein expression in 16HBE cells treated with CSE, FHL1 knockdown, and MAPK pathway inhibitor SB203580 co-treatment. Data are presented as mean ± SD (*n* = 3). *P < 0.05

In elucidating the intricate mechanisms of COPD pathogenesis, our investigation centered on the modulation of the MAPK pathway, a quintessential element in inflammatory and apoptotic processes. Initial observations confirmed that exposure to CSE markedly increased the levels of p-MEK1 and p-p38 MAPK in 16HBE cells, signaling an activation of the MAPK cascade (Fig. 7A). Intriguingly, overexpression of SNHG4 markedly dampened this activation, implicating a regulatory control over key signaling molecules [30]. Further probing into the mechanism revealed that SNHG4’s regulatory effect is mediated through the miR-409-3p/FHL1 axis. The knockdown of FHL1 notably reversed the inhibitory effects of SNHG4 on the phosphorylation of MEK1 and p38 MAPK, reinstating the activation of the MAPK pathway (Fig. 7B). This dynamic was further validated by treating cells with FHL1 knockdown and observing a significant upregulation in the expression of p-MEK1 and p-p38 MAPK, compared to controls (Fig. 7C). Notably, the addition of the MAPK pathway inhibitor SB203580 to the cellular milieu elucidated the potential for targeted therapeutic intervention, demonstrating that the inhibition of the MAPK pathway could effectively counteract the enhanced expression of p-MEK1 and p-p38 MAPK induced by FHL1 knockdown (Fig. 7D). Collectively, these findings articulate a sophisticated regulatory narrative where SNHG4, through its interaction with the miR-409-3p/FHL1 axis, exerts a potent modulatory effect on the MAPK pathway.

### Enhanced COPD pathological manifestations in mice following SNHG4 knockdown

**Fig. 8:**
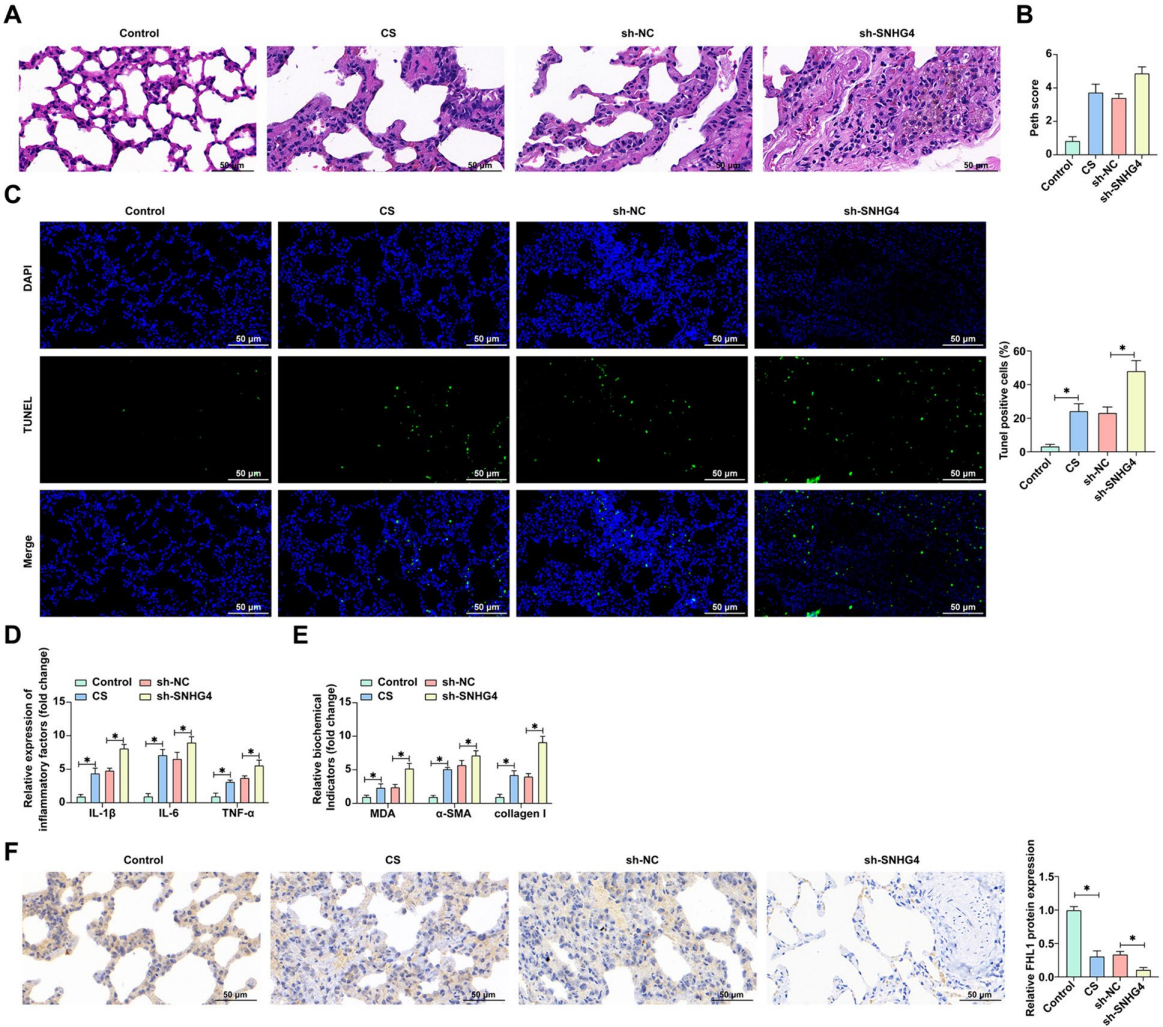
Knockdown of SNHG4 exacerbates pathological damage in COPD mice. **A** Representative images of mouse lung tissues stained with HE (×40); **B** functional assessment of mouse lung tissues using Penh score; **C** representative images of TUNEL staining for apoptosis in mouse lung tissues (×40); **D** ELISA measures the levels of TNF-α, IL-1β, IL-6 in mouse lung tissues BALF; **E** ELISA and commercial kits assess the levels of MDA, α-SMA, collagen I, SOD in mouse lung tissues; **F** IHC staining detects FHL1 expression in lung tissues (×40). Data are presented as mean ± SD (*n* = 6). *P < 0.05

In an effort to bridge our in vitro findings with in vivo phenomena, we utilized a COPD mouse model subjected to targeted SNHG4 knockdown. Histopathological examination revealed that SNHG4 knockdown significantly exacerbated lung tissue congestion, parenchymal consolidation, and inflammatory infiltration in COPD mice, delineating a clear trajectory of worsened pathology (Fig. 8A). Additionally, the Penh scores of COPD mice were significantly higher than those of the control group, and knockdown of SNHG4 further increased the Penh scores (Fig. 8B). Complementary TUNEL staining further confirmed the increased apoptotic rate in lung tissues post-SNHG4 reduction, indicating its vital role in cellular survival within the COPD context (Fig. 8C). Biochemical evaluations of BALF from these mice showed a marked increase in inflammatory markers TNF-α, IL-1β, and IL-6 following SNHG4 knockdown, suggesting an elevated inflammatory response. Additionally, analysis of lung tissues revealed that SNHG4 reduction notably amplified the levels of pro-inflammatory cytokines and oxidative stress markers, while also accelerating the fibrotic remodeling, as evidenced by increased α-SMA and collagen I levels (Fig. 8D, E). Immunohistochemical analysis further demonstrated a significant decrease in FHL1 expression in COPD mice, with an even more pronounced reduction post-SNHG4 knockdown (Fig. 8F). It is noteworthy that the knockdown of SNHG4 did not induce lung injury in healthy mice, suggesting that the role of SNHG4 may be more pertinent under specific stimulus conditions, such as exposure to tobacco smoke (Additional file 1: Fig. S1). These findings collectively underscore the critical role of SNHG4 in modulating the complex pathological landscape of COPD, where its knockdown leads to exacerbated inflammation, apoptosis, and fibrotic remodeling, hence worsening the disease’s pathological impact.

## Discussion

COPD, a principal cause of morbidity and mortality globally, exerts significant impacts on public health and quality of life. Yet, effective and specific therapies for COPD remain elusive. Emerging evidence indicates that lncRNAs are aberrantly expressed in COPD, potentially modulating protein-coding gene expression through cis or trans regulatory mechanisms, implicating their role in the pathogenesis of COPD [31, 32]. For instance, lncRNA MIR155HG is reported to facilitate apoptosis and inflammation in smoke-related COPD via targeting the miR-128-5p/BRD4 axis [33]. Additionally, interactions between differentially expressed lncRNAs and miRNAs offer novel avenues for identifying therapeutic targets in COPD [34]. This study aims to elucidate the expression, biological function, and regulatory mechanisms of lncRNASNHG4 in COPD. We demonstrate for the first time the biological role of lncRNASNHG4 in COPD, where it ameliorates CSE-induced apoptosis, inflammation, oxidative stress, and airway remodeling by competitively binding miR-409-3p to modulate FHL1 expression. These effects are associated with the suppression of MAPK signaling activation.

Exposure to CSE can trigger abnormal inflammatory responses in bronchioles and alveoli, hastening apoptosis of bronchial epithelial cells [35]. CSE-treated bronchial epithelial cells are widely used as an in vitro model for COPD [36]. Thus, this study employs CSE-induced 16HBE cells as an in vitro model for COPD. SNHG4, a well-studied lncRNA, is found to be downregulated in neonatal pneumonia and attenuates lipopolysaccharide-induced inflammatory lung injury by inhibiting METTL3-mediated m6A modification of STAT2 mRNA [11]. We discovered significant downregulation of SNHG4 in COPD tissues and CSE-induced 16HBE cells. Moreover, upregulation of SNHG4 diminishes the anti-proliferative and pro-apoptotic effects of CSE on 16HBE cells. Additionally, knocking down SNHG4 in a COPD mouse model exacerbates lung inflammation, oxidative stress, and apoptosis. Given that inflammation is a key factor in lung disease and airflow limitation in COPD [37], reducing inflammatory cytokines by suppressing inflammation and airway remodeling has beneficial effects on COPD progression.

Accumulated evidence suggests the lncRNA–miRNA–mRNA signaling regulatory network plays a critical role in COPD progression [38]. Moreover, reports have indicated that SNHG4 competitively binds miRNA, mitigating miRNA-mediated post-transcriptional repression of target mRNAs, thus participating in disease-related biological processes. For instance, SNHG4 involves in NSCLC progression through the miR-let-7e/KDM3A/p21 pathway [18]. Our study identified miR-409-3p as a downstream target of SNHG4 through bioinformatics prediction, dual-luciferase reporter assay, and RIP experiments. miR-409-3p, implicated in various lung diseases, including pneumonia [15] and lung cancer [39, 40], is found to be upregulated in COPD. Overexpression of miR-409-3p reverses the ameliorative effects of SNHG4 upregulation on CSE-induced 16HBE cell proliferation, apoptosis, inflammation, and airway remodeling.

To further explore the downstream mechanism of miR-409-3p, we conducted bioinformatics analysis and predicted FHL1 as a potential target. FHL1, belonging to the four and a FHL family, regulates proliferation, differentiation, apoptosis, adhesion, migration, and other cellular processes [41]. Reported to be downregulated in COPD, FHL1 is closely associated with CSE-induced COPD [16]. As anticipated, we found FHL1 downregulated in COPD, and its expression can be restored by downregulating miR-409-3p. Importantly, the downregulation of FHL1 offsets the beneficial effects of SNHG4 on CSE-treated HBE cells, indicating SNHG4 mediates COPD progression by targeting the miR-409-3p/FHL1 axis.

The MAPK signaling pathway, a prevalent inflammatory signaling pathway, modulates disease conditions [42]. Activation of the MAPK pathway is reported to exacerbate COPD pathologies. For example, overexpressing FOXA2 mitigates CSE-induced cellular damage and pulmonary inflammation by inhibiting the p38 and Erk1/2 MAPK pathways [43]. Similarly, ACE2 reduces COPD inflammation by lowering oxidative stress and inhibiting NF-κB and p38 MAPK pathway activation [44]. In this study, we discovered that SNHG4 impedes the activation of the MAPK pathway induced by CSE through the upregulation of FHL1. This process is likely a crucial pathway for ameliorating inflammation and apoptosis in alveolar epithelial cells.

In conclusion, our research confirms that SNHG4, by targeting the miR-409-3p/FHL1 axis, regulates the MAPK signaling pathway, mitigating CSE-induced cellular proliferation, apoptosis, inflammation, and airway remodeling in 16CSE cells. The beneficial effects of SNHG4 on COPD have also been validated in animal studies, offering new insights for targeted COPD therapy. However, the clinical application of SNHG4 necessitates further validation in clinical studies, and due to interspecies differences, the clinical translation of SNHG4 in COPD remains a significant challenge. Additionally, while this study focused on bronchial epithelial cells and epithelial cells, further investigation is needed to determine the impact of SNHG4 on lung tissue cells and immune cells.

### Supplementary Information

**Additional file 1: Figure S1.** Knockdown of SNHG4 does not induce lung injury in healthy mice. A: Pulmonary architecture in control and SNHG4-silenced healthy mice was conducted using HE staining; B: Investigation of apoptotic cell death within the pulmonary tissues of healthy mice following SNHG4 knockdown was performed via TUNEL) staining (*n* = 6).

**Additional file 2: Table S1.** sh-FHL1 sequences.

## Data Availability

Not applicable.
