# Supplementary figures and images for: Long non-coding RNA Small Nucleolar RNA Host Gene 4 ameliorates cigarette smoke-induced proliferation, apoptosis, inflammation, and airway remodeling in alveolar epithelial cells through the modulation of the mitogen-activated protein kinase signaling pathway via the microRNA-409-3p/Four and a Half LIM Domains 1 axis
Source: Eur J Med Res. 2024 Jun 4;29:309. doi: 10.1186/s40001-024-01872-x (PMC11149209; doi:10.1186/s40001-024-01872-x)

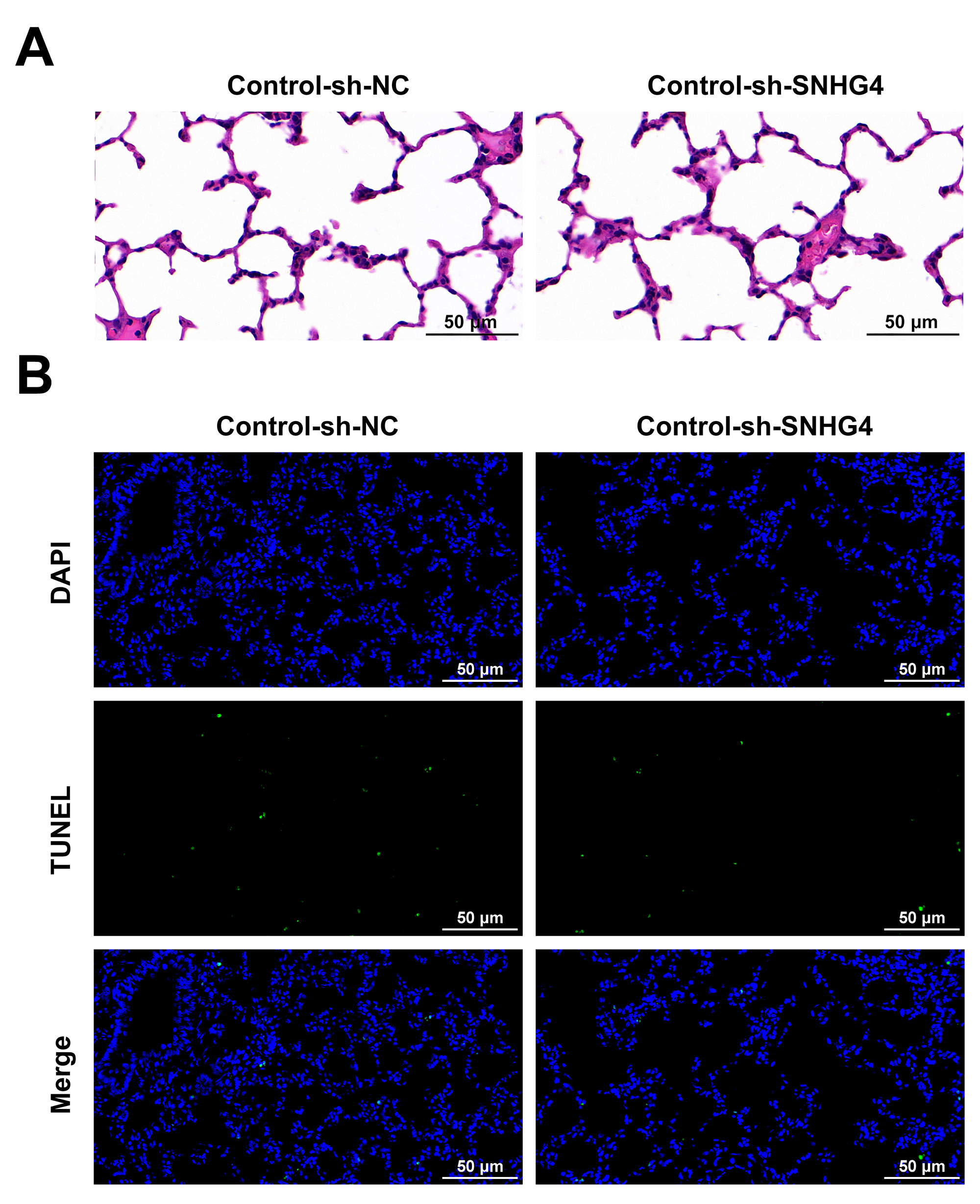

Supplement: Supplementary file 1 — Additional file 1: Figure S1. Knockdown of SNHG4 does not induce lung injury in healthy mice. A: Pulmonary architecture in control and SNHG4-silenced healthy mice was conducted using HE staining; B: Investigation of apoptotic cell death within the pulmonary tissues of healthy mice following SNHG4 knockdown was performed via TUNEL) staining (n = 6). [file 40001_2024_1872_MOESM1_ESM.tif]
