# Supplementary material for: Long non-coding RNA Small Nucleolar RNA Host Gene 4 ameliorates cigarette smoke-induced proliferation, apoptosis, inflammation, and airway remodeling in alveolar epithelial cells through the modulation of the mitogen-activated protein kinase signaling pathway via the microRNA-409-3p/Four and a Half LIM Domains 1 axis
Source: Eur J Med Res. 2024 Jun 4;29:309. doi: 10.1186/s40001-024-01872-x (PMC11149209; doi:10.1186/s40001-024-01872-x)
Supplement: Supplementary file 2 — Additional file 2: Table S1. sh-FHL1 sequences. [file 40001_2024_1872_MOESM2_ESM.docx]

**Table S1** sh-FHL1 sequences

| shRNA | Target position | Primer sequences (5'–3') |
| --- | --- | --- |
| Sh-FHL1#1 | 245-267 | 5'-AAGAAGTATGTGCAAAAGGATGG-3' |
| Sh-FHL1#2 | 1037-1059 | 5'-GTGGTTCTTTATAGAAAAAATCG-3' |
| Sh-FHL1#3 | 1041-1063 | 5'-TTCTTTATAGAAAAAATCGAAGC-3' |
